# Intermittent Fasting Results in Tissue-Specific Changes in Bioenergetics and Redox State

**DOI:** 10.1371/journal.pone.0120413

**Published:** 2015-03-06

**Authors:** Bruno Chausse, Marcel A. Vieira-Lara, Angélica B. Sanchez, Marisa H. G. Medeiros, Alicia J. Kowaltowski

**Affiliations:** Departamento de Bioquímica, Instituto de Química, Universidade de São Paulo, São Paulo, Brazil; University of Tasmania, AUSTRALIA

## Abstract

Intermittent fasting (IF) is a dietary intervention often used as an alternative to caloric restriction (CR) and characterized by 24 hour cycles alternating *ad libitum* feeding and fasting. Although the consequences of CR are well studied, the effects of IF on redox status are not. Here, we address the effects of IF on redox state markers in different tissues in order to uncover how changes in feeding frequency alter redox balance in rats. IF rats displayed lower body mass due to decreased energy conversion efficiency. Livers in IF rats presented increased mitochondrial respiratory capacity and enhanced levels of protein carbonyls. Surprisingly, IF animals also presented an increase in oxidative damage in the brain that was not related to changes in mitochondrial bioenergetics. Conversely, IF promoted a substantial protection against oxidative damage in the heart. No difference in mitochondrial bioenergetics or redox homeostasis was observed in skeletal muscles of IF animals. Overall, IF affects redox balance in a tissue-specific manner, leading to redox imbalance in the liver and brain and protection against oxidative damage in the heart.

## Introduction

Moderate reduction in *ad libitum* daily caloric intake without limiting essential and micronutrient access (caloric restriction, CR) is a well-established mechanism to promote longer lifespans and/or healthier aging in a wide range of organisms, including humans [1, 2]. The beneficial effects of CR diets are complex and widespread, but include the preservation over time of mitochondrial bioenergetic functions and redox balance (the difference between the production and removal of damaging oxidative species) [3, 4, 5].

The downside of CR is that it requires daily control of caloric intake, and is thus labor-intensive both for humans and to maintain in a laboratory animal setting. As an alternative to CR, some groups have proposed the use of intermittent fasting (IF), also referred to as intermittent feeding or every other day feeding [6, 7], in which *ad libitum* feeding periods are alternated with fasting, thus avoiding the strict daily control of caloric intake. In laboratory animals, this feeding protocol was first described by Carlson and Hoelzel, who observed a slight increment in rat life span [8]. Despite this early result suggesting a lifespan benefit similar to CR, subsequent studies have indicated that the enhancement in life expectancy promoted by IF, if present, is certainly less robust [9, 10]. In humans, IF has been shown to reduce obesity and have positive effects on some pathologies [11, 12].

Despite some similarities such as promoting reduced body weight [13], substantial differences between CR and IF have been uncovered [5] and are, indeed, expected, since CR involves a daily reduction in food intake, while IF changes feeding frequency, and may not always result in a limitation of food ingestion [7, 14]. In fact, while CR enhances insulin sensitivity, we find that long-term IF results in insulin resistance [13]. We also find that long-term IF enhances tissue oxidant release and oxidative protein modifications in the skeletal muscle and adipose tissue [13], while long-term CR is mostly associated with the prevention of oxidative damage [4, 5]. Overall, IF effects on oxidative balance are not yet well determined. This study bridges this knowledge gap, and demonstrates that short-term IF affects redox balance in a tissue-selective manner.

## Materials and Methods

### Animals

Experiments were conducted in agreement with the NIH guidelines for the humane treatment of animals and were approved by the local Animal Care and Use Committee (Comissão de Ética em Cuidado e Uso Animal—Instituto de Química, USP) (Permit number: 21/2014). Male, 8-week-old Sprague Dawley rats were separated in two groups: one fed *ad libitum* (AL), and one with *ad libitum* access to food on alternating days (IF). The animals were lodged four individuals per cage in 12 hours light/dark cycles and given water *ad libitum*. Body mass was recorded before and after 1 month of the dietary intervention and cumulative food consumption over the treatment period was recorded for each cage. The average intake per animal was used to calculate feed conversion efficiency (average weight gain per cage × 100/average food intake per cage). Chow was alternately placed or removed at 5:00 pm for IF animals. After 1 month of the dietary intervention, AL and IF rats were euthanized by decapitation after 12 hours of overnight fasting.

### Mitochondrial isolation

Mitochondrial isolation from different tissues was performed as described by Tahara et al., 2009 [15]. Briefly:

**Liver mitochondria**. The tissue was minced finely and washed with 4°C isolation buffer (250 mM sucrose, 10 mM Hepes, 1 mM EGTA, pH 7.2) and then homogenized with a 40 mL tissue grinder. The suspension was centrifuged at 600 g for 5 minutes and the resulting supernatant was centrifuged at 12,000 g for 5 minutes. The pellet was washed and resuspended in a minimal volume of the same buffer.

**Brain mitochondria**. The preparation used digitonin to release mitochondria from synaptosomes, resulting in a mixture of synaptosomal and nonsynaptosomal mitochondria. The brain was minced and washed with 125 mM sucrose, mannitol 250 mM, 10 mM Hepes, 10 mM EGTA, 0.01% BSA, pH 7.2 at 4°C. The homogenate was centrifuged at 2,000 g for 3 minutes and the supernatant transferred to a new tube and centrifuged at 12,000 g for 8 minutes. The resulting pellet was resuspended in a minimal volume of the isolation buffer devoid of EGTA.

**Skeletal muscle mitochondria**. Hind-limb muscles were dissected in isolation buffer (300 mM sucrose, 50 mM Hepes, 10 mM Tris, 1 mM EGTA, 0.2% BSA, pH 7.2) at 4°C to remove fatty and connective tissue. The tissue was processed with a Polytron homogenizer and then with a mechanized potter. The homogenate was centrifuged at 850 g for 5 minutes and the supernatant was centrifuged again at 10,000 g for 5 minutes. The pellet was resuspended and centrifuged at 7,000 g for 5 minutes. The final mitochondrial pellet was prepared in a minimal volume of isolation buffer.

**Heart mitochondria**. Hearts were minced finely in isolation buffer (300 mM sucrose, 10 mM Hepes, 2 mM EGTA, pH 7.2) at 4°C, in the presence of 0.5 μM type I protease (bovine pancreas) to release mitochondria from the muscle fibers, and then washed in the same buffer in the presence of 1 mg/mL BSA. The suspension was homogenized in a 40 mL tissue grinder and centrifuged at 800 g for 5 minutes. The resulting supernatant was centrifuged at 9,500 g for 10 minutes and the mitochondrial pellet was resuspended in a minimal amount of isolation buffer.

### Mitochondrial H_2_O_2_ release

Mitochondrial H_2_O_2_ release was measured in 0.125–0.500 mg protein/mL mitochondrial suspensions in experimental buffer (150 mM KCl, 10 mM Hepes, 2 mM KH_2_PO_4_, 0.1% BSA, pH 7.2) at 37°C, with continuous stirring in the presence of 25 μM Amplex Red and 0.5 U/mL horseradish peroxidase (HRP). Malate/glutamate, pyruvate/malate or succinate were used as substrates. Amplex Red is oxidized in the presence of extramitochondrial horseradish peroxidase bound to H_2_O_2_, generating resorufin, which can be detected with a fluorescence spectrophotometer operating at 563 nm excitation and 587 nm emission [16].

### Mitochondrial O_2_ consumption

O_2_ consumption was monitored using a Clark-type electrode, coupled to a high-resolution Oroboros respirometry system [17] in 0.125–0.500 mg protein/mL mitochondrial suspensions under the same buffering conditions used in the H_2_O_2_ assay.

### Citrate synthase activity

Tissues were homogenized with an electric potter in 50 mM phosphate buffer (pH, 7.5) at 4°C and centrifuged at 600 g for 10 minutes, in the presence of protease inhibitor (1:100). A mixture of 100 mM Tris-HCl, 100 μM Acetyl-CoA, 100 μM DTNB, 250 μM oxaloacetate and 0.1% Triton-X, pH 8.1, was incubated with homogenate supernatant at 30°C for 5 minutes. CoA-SH produced by the enzymatic reaction reduces the DTNB, which is followed by the increase in absorbance at 412 nm (extinction coefficient = 13.6 mM^-1^·cm^-1^) (adapted from ref [18]).

### Immunoblots

**COX4 and NRF-1**. Liver homogenates were prepared under the same conditions used for the citrate synthase assay and then diluted 4:1 in Laemmli buffer (100 mM Tris-HCl, 2% SDS, 10% glycerol and 0.1% bromophenol blue) for NRF-1 determinations. For COX4, homogenates were diluted in the same buffer, and heated at 100°C for 10 minutes. The proteins were separated by SDS-PAGE and transferred to nitrocellulose (COX4) or PVDF (NRF-1) membranes, which were then incubated with 5% BSA. Chemiluminescent detection was performed using primary anti-COX4 (1:1000), anti-NRF-1 (1:1000) antibodies and the respective secondary anti-mouse IgG (1:5000) and anti-rabbit IgG (1:5000) antibodies, both bound to peroxidase. The signals were quantified using ImageJ software and normalized by coloring the nitrocellulose and PVDF membranes with Ponceau and Coomassie Blue, respectively.

**Carbonylated Proteins**. Tissue homogenates were prepared under the same conditions as the citrate synthase assay. A derivatization of the sample was performed, adding equal volumes of 24% SDS and 40 mM 2,4-dinitrophenylhydrazine (DNPH, prepared in 10% trifluoroacetic acid). The mixture, incubated in the dark for 30 minutes, allows the reaction between the DNPH and carbonyl groups in proteins. Samples were then treated with a neutralizing solution (1:3), of 2 M Tris, 30% glycerol and 19% 2-mercaptoethanol. The proteins were separated by SDS-PAGE and transferred to a PVDF membrane (adapted from ref. [19]).

**Nitro-tyrosin (NO_2_-Try) levels**. Tissue homogenates were prepared under the same conditions as described for the citrate synthase assay. For dot blot experiments, 10 μL of homogenates containing 3 μg of protein were placed on nitrocellulose membranes. After they were dry, the membranes were blocked in a 4% BSA solution for one hour followed by an overnight incubation with primary anti-NO_2_-Try (1:2000). After one hour incubation with the anti-rabbit IgG (1:10000) secondary antibody, NO_2_-Try signals were identified using an Odyssey CLx system (LI-COR, Nebraska, USA). The signals were quantified using ImageJ software and normalized to protein levels by coloring the nitrocellulose membranes with Ponceau.

### Antioxidant enzyme activities

Tissue homogenates were prepared as described for the citrate synthase assay. Glutathione peroxidase (GSH-Px) activity was measured as described in ref. [20]. A mixture of phosphate buffer, reduced glutathione (GSH), glutathione reductase (GR), EDTA, NADPH, NaN_3_ and homogenate was used, and the reaction was initiated by the addition of H_2_O_2_. The decrease in the quantity of NADPH was followed by absorbance at 340 nm for 3 minutes. The glutathione reductase assay followed the protocol described in ref. [21]. GR catalyzes the reduction of GSSG to GSH in the presence of NADPH. The resulting GSH reacts with 5,5′-dithio-bis-2-nitrobenzoic acid (DTNB), producing 5-thio-2-nitrobenzoic acid (TNB), detected by absorbance at 412 nm. The catalase assay was adapted from ref. [22]. Homogenates were incubated in the same experimental buffer used in the H_2_O_2_ assay and the O_2_ production rate was measured for 30 seconds after the addition of 200–800 μM H_2_O_2_ using a Clark-type electrode at 37°C with continuous stirring.

### Glutathione levels

The concentrations of GSH and GSSG, as well as total glutathione, were determined using a colorimetric assay with DTNB, as described in ref. [23]. GSH reacts with DTNB producing TNB and GS-TNB, which is reduced back to GSH by GR. This process releases TNB, detected by absorbance at 412 nm.

### Malondialdehyde (MDA) determination

Tissues were homogenized in TKM buffer (50 mM Tris-HCl, 25 mM KCl and 5 mM MgCl_2_ and SDS 10%, pH 7,5). One milliliter of thio-barburic acid (TBA; 0.4% w/v in 133 mM HCl) and 150 uL butylated-hydroxytoluene (BHT; 0.2% w/v in 95% ethanol) were added to 1.2 mL of the homogenate. The mixture was heated to 90°C for 45 min, cooled on ice, and extracted with isobutanol. The isobutanolic phase was injected through a Shimadzu auto injector model SIL-20A (Shimadzu, Kyoto, Japan) in a Shimadzu HPLC system consisting of two pumps; LC-20AT connected to a Luna C18 (Phenomenex, Torrance, CA) reversed-phase column (250 mm x 4.6 mm i.d., particle size 10 μm). The flow rate of the isocratic eluent (5 mM potassium phosphate buffer, pH 7, with 23% methanol) was 1 mL/min. A RF-10A/XL fluorescence detector was set at an excitation wavelength of 515 nm and an emission wavelength of 553 nm. The data were processed using the Shimadzu LC Solution 1.25 software. Malonaldehyde-bisdiethylacetal was used to calibrate the fluorescence data, yielding a quantitative adduct of the malonaldehyde-TBA product. The data were expressed as pmol of MDA normalized by protein mass (adapted from [24]).

### Statistical analysis

Data were analyzed using GraphPad Prism and Origin softwares. Figures are presented as average ± standard error and comparisons were performed using two-tailed Student t-tests. p<0.05 was considered significant.

## Results

### IF reduces body mass and energy conversion efficiency

**Fig 1:**
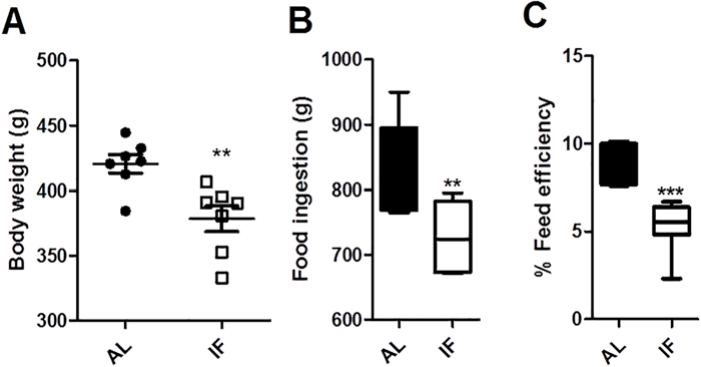
Intermittent fasting promotes lower body mass related to a mild reduction in caloric intake and lower energy conversion efficiency. (A) Average body weight per cage in (●) AL and (☐) IF animals after one month of treatment. (B) Cumulative food intake over one month of treatment. (C) Energy conversion efficiency. Data represent averages ± SEM and were compared using *t* tests (n = 7 cages). ** p<0.01 vs AL. AL indicates *ad libitum* feeding, IF indicates intermittent fasting.

IF diets have been extensively shown to reduce body mass [6, 7, 9]. Indeed, we observed that, after a month, IF rats presented lower body weights than AL control rats (Fig. 1A). The decrease in body weight was a reflection of two additive effects of the diet: a 14% reduction in food consumption (Fig. 1B) and a 40% decrease in energy conversion efficiency (Fig. 1C), or the amount of food ingested that results in weight gain. Similar decreases in energy conversion efficiency in IF have been reported previously and may be related to changes in hypothalamic energy metabolism control [25].

### IF increases respiratory capacity in the liver, but not in other tissues

Restricted diets have been extensively shown to be related to changes in mitochondrial bioenergetics [2, 3, 26] which in turn are known to be determinant toward redox balance, since mitochondria are the most quantitatively relevant source of intracellular reactive oxygen species (ROS). We thus sought to determine if short-term IF lead to changes in mitochondrial bioenergetics in different tissues.

**Fig 2:**
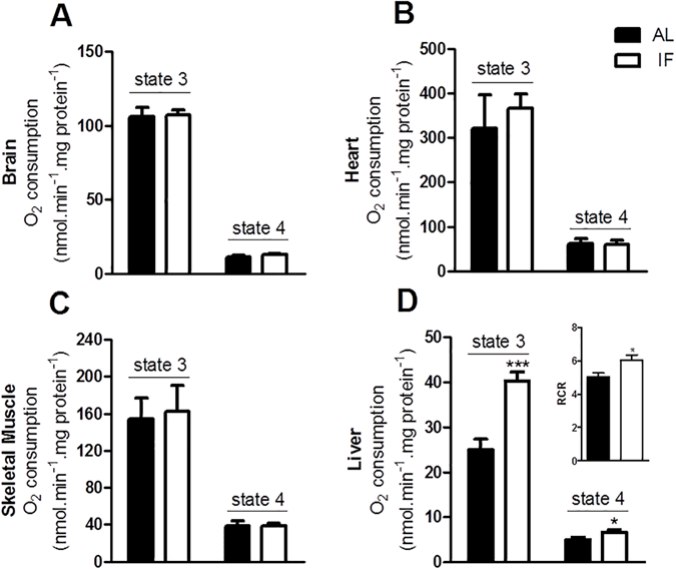
IF induces an increase in respiratory capacity in liver, without bioenergetic changes in other tissues. Oxygen consumption in isolated mitochondria from (A) brain, (B) heart, (C) skeletal muscle and (D) liver in the presence of 5 mM pyruvate plus 3 mM malate (A, B and D) or 2 mM glutamate plus 2 mM malate (C). State 3 was induced by the addition of 1 mM ADP and state 4 was achieved using 0.5 μg/mL oligomycin. The insert in Panel D represents respiratory control ratios (RCR), or state 3/state 4. Data represent averages ± SEM and were compared using *t* tests (n = 4–6 animals). * p<0.05 vs AL in the same respiratory state.

In Fig. 2, we measured isolated mitochondrial NADH-linked oxygen consumption rates using samples obtained from different tissues under two conditions: state 3, in which ADP is present, stimulating maximal ATP synthesis-linked respiration; and state 4, in which the ATP synthase inhibitor oligomycin is present, and respiration reflects the magnitude of the proton leak [27]. Interestingly, respiratory rates in both states were unchanged by IF in the brain (Fig. 2A), heart (Fig. 2B) and skeletal muscle (Fig. 2C). On the other hand, both ADP-stimulated and oligomycin-inhibited respiratory rates were increased in liver mitochondria isolated from IF animals (Fig. 2D). This increase in respiratory rate was accompanied by a slight increase in respiratory control ratios (the ratio between state 3 and state 4 oxygen consumption) in IF samples, indicating that it reflects an increment in electron transfer capacity and is not a consequence of increased proton leak.

**Fig 3:**
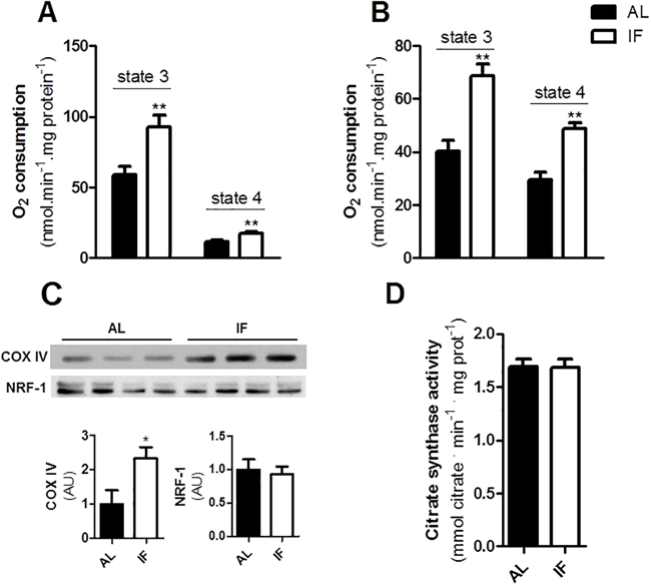
IF promotes enhanced electron transport capacity in the liver. Oxygen consumption in isolated mitochondria from livers in the presence of (A) 1 mM succinate plus 1 μM rotenone or (B) 200 μM TMPD plus 2 mM ascorbate. State 3 and state 4 were induced by ADP and oligomycin as described for Fig. 2. (C) COX-IV and NRF-1 levels measured as described in Materials and Methods. (D) Liver citrate synthase activity, determined as described in Materials and Methods. Data represent averages ± SEM and were compared using *t* tests (4–6 animals). * p<0.05, ** p<0.01 vs AL.

In order to further investigate the effects of IF on liver mitochondrial bioenergetics, we determined respiratory rates using succinate (which feeds electrons into the respiratory chain through Complex II; Fig. 3A) and TMPD-ascorbate (which reduces cytochrome c and cytochrome c oxidase; Fig. 3B). Both substrates also supported higher oxygen consumption rates in samples from IF animals, indicating that the enhanced respiratory capacity involves the enhanced activity of terminal electron carriers. Indeed, tissue levels of mitochondrial complex IV (cytochrome oxidase, COX, Fig. 3C) were significantly enhanced in IF livers.

The activity of the mitochondrial electron transport chain is often linked to an overall increase in the biogenesis of all mitochondrial enzymes. To test if this was the case, we determined the levels of NRF-1, a transcription factor that activates key mitochondria biogenesis, promoting mitochondrial DNA transcription and replication. Interestingly, NRF-1 levels were unaltered by IF (Fig. 3C). Confirming that liver mitochondrial biogenesis as a whole was not induced by IF, we found that activity of citrate synthase, a citric acid cycle enzyme and mitochondrial mass marker was equal in both groups (Fig. 3D). Altogether, these data suggests that IF induces an enrichment in respiratory chain proteins in liver mitochondria, without an overall increase in mitochondrial mass.

### Mitochondrial reactive oxygen species release is unaltered by IF

**Fig 4:**
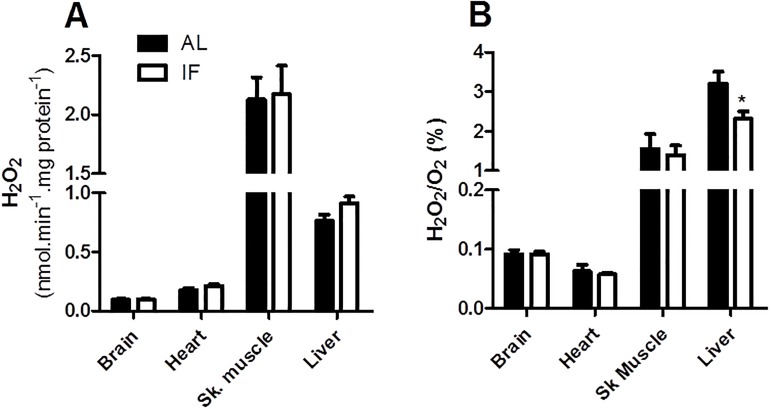
Reactive oxygen species production is not significantly altered by IF. (A) H_2_O_2_ release by isolated mitochondria in the presence of 1 mM ADP (state 3) and 5 mM pyruvate plus 3 mM malate (brain, heart and liver) or 2 mM glutamate plus 2 mM malate (skeletal muscle). (B) Ratio between H_2_O_2_ production and O_2_ consumption by isolated mitochondria under the same conditions as panel A. Data represent averages ± SEM and were compared using *t* tests (n = 4–6 animals).

In addition to determining changes in functional parameters, we measured the release of mitochondrial H_2_O_2_, a relatively stable and membrane-permeable ROS often used as a marker for oxidant production (Fig. 4) [28]. Interestingly, we found that IF did not significantly alter absolute H_2_O_2_ release in any tissue (Fig. 4A), although liver release was slightly increased (p = 0.07). IF decreased relative H_2_O_2_/O_2_ release (Fig. 4B) in liver due to the enhanced O_2_ consumption observed in Figs. 2 and 3, and no change in this ratio was observed in other tissues. Since changes in respiratory rates and mitochondrial coupling are often determinant toward mitochondrial oxidant generation, this result is compatible with our finding that IF does not alter mitochondrial bioenergetic parameters in brain, heart and skeletal muscle. Furthermore, the lack of changes in H_2_O_2_ in liver are compatible with the prior finding that oxidant release in this tissue is not strongly regulated by respiratory rates and coupling [15].

### IF effects on antioxidants and oxidative damage are tissue-specific

Tissue redox status depends not only on the production of ROS but also on the levels and activities of antioxidant systems. We thus measured the activities of several key antioxidant enzymes in IF versus AL tissues (Table 1). The activity of glutathione peroxidase (GPx) [29], which uses glutathione (GSH) as an electron source to reduce and remove H_2_O_2_, was significantly decreased by IF in the skeletal muscle, and unchanged in all other tissues. Glutathione reductase, which restores GSH, was unchanged by this dietary intervention, in all tissues. Catalase, which also removes H_2_O_2_, was decreased by IF in the brain and unchanged in the other tissues. Overall, our data indicate that IF does not increase antioxidant enzyme activity and, if anything, promotes some tissue-specific decreases in oxidant buffering capacity.

**Table 1:** Antioxidant enzyme activities.

		Brain	Heart	Muscle	Liver
**Glutathione Peroxidase**	**AL**	22.6 ± 1.30	119.9 ± 9.94	238.2 ± 13.13	168.5 ± 29.29
(mU^·^ mg prot^-1^)	**IF**	21.2 ± 0.93	91.5 ± 8.52	195.0 ± 11.50*	174.8 ± 9.65
**Glutathione Reductase**	**AL**	5.21 ± 0.16	4.52 ± 0.34	12.80 ± 0.49	11.89 ± 0.26
(mU^·^ mg prot^-1^)	**IF**	5.48 ± 0.09	4.04 ± 0.34	12.48 ± 0.55	12.02 ± 0.26
**Catalase**	**AL**	0.281 ± 0.02	0.933 ± 0.18	4.742 ± 0.36	0.841 ± 0.05
(μmol^·^ mg prot^-1·^ min^-1^)	**IF**	0.229 ± 0.01*	0.850 ± 0.17	4.954 ± 0.39	0.738 ± 0.10

Values are means ± SEM and were compared using *t* tests (n = 4 animals).

* p<0.05 vs AL. AL indicates *ad libitum* feeding, IF indicates intermittent fasting.

We also determined the levels of different forms of glutathione, an intracellular reductant and redox state marker (Table 2). Levels of total glutathione (reduced plus oxidized), GSH, oxidized glutathione (GSSG) and GSH/GSSG ratios were mostly unchanged by IF, with a few exceptions: Livers of IF rats presented a significant increase in total glutathione and reduced GSH contents, which may be a consequence of a chronic exposure to a slightly increased oxidant generation (Fig. 4A). On the other hand, IF hearts presented lower levels of GSSG and, consequently, elevated GSH/GSSG ratios (Table 2), an indicative of a more reduced intracellular environment.

**Table 2:** Glutathione levels.

		Brain	Heart	Muscle	Liver
**Total Glutathione**	**AL**	40.96 ± 3.06	21.24 ± 1.02	68.36 ± 8.19	11.37 ± 0.39
(nmol^·^ mg protein^-1^)	**IF**	50.69 ± 2.81	22.63 ± 0.86	80.61 ± 14.57	15.30 ± 0.86*
**GSH**	**AL**	35.06 ± 3.42	14.57 ± 1.81	44.51 ± 8.76	9.00 ± 0.78
(nmol^·^ mg protein^-1^)	**IF**	42.68 ± 2.45	19.44 ± 0.92	53.95 ± 13.36	12.70 ± 0.43*
**GSSG**	**AL**	3.62 ± 0.42	3.34 ± 0.42	11.92 ± 1.97	1.18 ± 0.20
(nmol^·^ mg protein^-1^)	**IF**	4.64 ± 0.32	1.60 ± 0.15*	13.33 ± 1.08	1.30 ± 0.21
**GSH/GSSG**	**AL**	9.00 ± 0.34	4.68 ± 1.24	4.25 ± 1.38	8.36 ± 2.22
	**IF**	9.04 ± 0.29	12.45 ± 1.60*	4.03 ± 0.89	10.29 ± 1.62

Values are means ± SEM and were compared using *t* tests (n = 3–4 animals).

* p<0.05 vs AL. AL indicates *ad libitum* feeding, IF indicates intermittent fasting.

**Fig 5:**
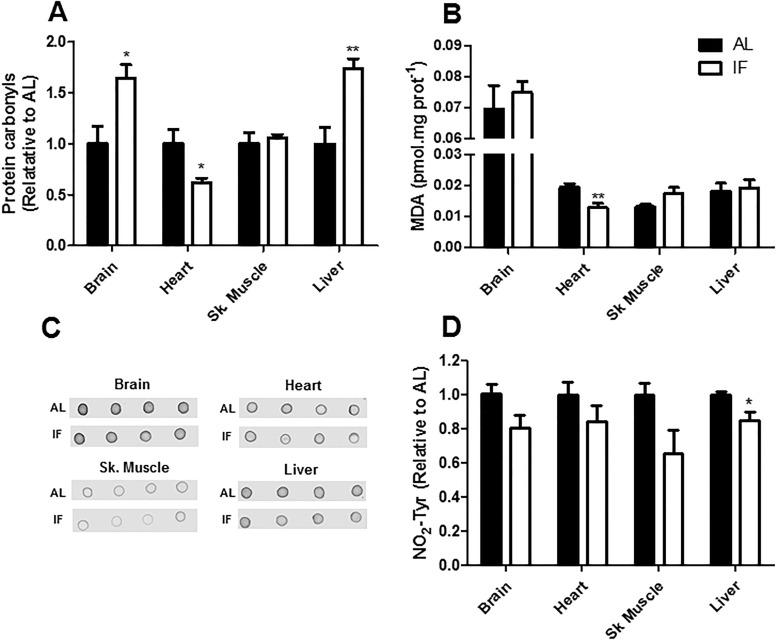
IF changes oxidative damage to biomolecules in a tissue-specific manner. (A) Carbonyl signals were quantified as described in Materials and Methods. (B) Malondaldehyde (MDA) levels were measured by HPLC as described in Materials and Methods. (C) Representative dot blots of NO_2_-tyr signals. (D) Average densitometric results for the dot blots presented in C. Data represent averages ± SEM and were compared using *t* tests (n = 4–5 animals). * p<0.05, ** p<0.01 vs AL.

To determine the consequences of these tissue-specific modifications in antioxidant capacity, we measured the levels of protein carbonyls, MDA (a product of lipoperoxidation reactions) and nitro-tyrosine (NO_2_-Tyr) as markers for biomolecule oxidative damage (Fig. 5). In keeping with the finding that IF hearts were in a more reduced state, as indicated by their GSSG levels (Table 2), we found that protein carbonyls and MDA levels were reduced by IF in this tissue (Fig. 5A and 5B). On the other hand, the brain presented enhanced protein carbonylation under IF, which may be a reflection of lower catalase activity. In the liver, a small decrease in the NO_2_-Tyr signal and a robust increase in protein carbonylation levels was observed. The protein carbonylation effect may be a consequence of the slightly enchanced in oxidant generation in this tissue. There was no dietary-promoted difference in the measured oxidative damage markers in the skeletal muscle. Altogether, these data suggest that short-term IF affects redox balance in a tissue-specific manner, promoting protection against oxidative damage in the heart and leading to enhanced protein carbonylation in the brain and liver.

## Discussion

Dietary interventions that reduce body weight relative to *ad libitum* food intake have received extensive attention due to their mostly positive effects on age-associated diseases [1, 2, 11]. Since aging is often accompanied by increased oxidative tissue damage and loss of mitochondrial activity, many studies involving CR have focused on the bioenergetic and redox effects of this diet [4, 30], mostly showing that it decreased oxidative damage [5]. On the other hand, IF is less studied, and sometimes even confused with CR in the literature [7], so its bioenergetic and redox effects are less clear. We thus sought to determine the redox and bioenergetic effects of IF and found that this diet affects these parameters in complex, tissue-specific manners.

Perhaps the most studied tissue relative to the effects of IF is the brain. Both IF and CR have been related to healthier brain aging by reducing oxidative damage [31–33]. Indeed, IF in aged animals partially restores the loss of some antioxidant enzymes, and decreases 8-hydroxy-deoxyguanosine DNA adducts accumulated over time [34, 35]. Thus, it was rather surprising to us to find that short-term IF in young animals leads to a significant increase in protein carbonyl signals in the brain of IF rats (Fig. 5), without measurable changes in mitochondrial function (Fig. 2), ROS release (Fig. 4), MDA or NO_2_-Tyr (Fig. 5). This increased protein oxidative damage may be related to a small but significant decrease in brain catalase activity promoted by IF (Table 1). How this early increase in brain oxidative modifications relates to neurological protection in long-term IF models still remains to be uncovered, but the effects are most probably unrelated, since other neuroprotective dietary interventions do not see increments in oxidative damage markers in the brain even in short-term studies [5].

IF and CR have also have been shown to protect the heart against ischemic injury [33, 36]. This protection has been attributed to activations in antioxidant defenses and stress response systems [35, 37]. In fact, long-term IF has been shown to improve antioxidant defenses and oxidative biomarker levels in the heart [34, 37, 38]. Here, we show that IF has an impact on heart redox state very early on, preventing the oxidation of glutathione (Table 2), protein carbonylation and MDA formation (Fig. 5).

Despite its central role in the control of energetic metabolism and aging, the skeletal muscle is still poorly studied regarding the effects of IF on redox state. Some long-term studies suggest that, unlike CR, IF may lead to a more oxidized state in this tissue, reducing antioxidant activity [39], increasing ROS production and oxidative protein modification [13]. Our short term study demonstrates that IF decreases glutathione peroxidase activity in the muscle (Table 1), although no changes are seen in glutathione redox state (Table 2) or oxidative damage markers (Fig. 5). Altogether, both our study and literature data suggest that IF does not prevent oxidative damage in the skeletal muscle and may actually worsen the redox condition of this tissue relative to *ad libitum* diets.

The liver is expected to be largely impacted by a dietary intervention such as IF, which alternates fasting periods with significant overeating. However, the few existent studies reporting IF effects on oxidative balance in the liver did not observe changes in antioxidant defense systems or in DNA and lipid damage [35, 38, 40]. Indeed, the same is observed for CR, in which most work indicates that there is no change in redox balance in the liver [5]. This finding may reflect the fact that ROS generation in the liver is not overtly modified by changes in metabolic conditions or respiratory substrates [15]. In this study, we find that short-term IF is actually detrimental toward liver oxidative balance, as reflected by the enhanced levels of protein carbonylation (Fig. 5) and induction of glutathione synthesis (Table 2), which may be related to a slight increment in mitochondrial H_2_O_2_ release (Fig. 4A). Again, this indicates that not all restrictive dietary interventions with positive health-span effects produce immediate more favorable redox conditions in all organs.

Interestingly, short-term IF had very significant bioenergetic effects in the liver that were not observed in other organs: it increased respiratory capacity, irrespective of the substrate used (Figs. 2, 3), but did not enhance overall mitochondrial biogenesis (Fig. 3). In a mouse model of IF in which more significant limitation in food intake occurred, IF was also found to increase liver respiratory capacity, but this effect was accompanied by mitochondrial biogenesis [3]. The increased electron transport capacity of IF livers may be important to deal with the large fluctuations in metabolic demands in the liver under this dietary regimen, which alternates fasting periods (characterized by gluconeogenesis and β oxidation) with overeating (which promotes liver glycolysis and fatty acid synthesis).

Overall, our studies show that IF promotes complex and tissue-specific changes in mitochondrial bioenergetics and tissue redox state in a manner that is distinct from that observed in other restrictive dietary interventions. How these changes relate to both the desirable and deleterious long-term results of this dietary intervention, and how they relate to human responses to repeated fasting cycles, remains to be determined.

## Supporting Information

S1 Dataset Raw data. (PDF)
