# Supplementary material for: Intermittent Fasting Results in Tissue-Specific Changes in Bioenergetics and Redox State
Source: PLoS One. 2015 Mar 6;10(3):e0120413. doi: 10.1371/journal.pone.0120413 (PMC4352038; doi:10.1371/journal.pone.0120413)
Supplement: S1 Dataset — (PDF) [file pone.0120413.s001.pdf]

Raw Data

Fig. 1A

AL	IF
444.5	406.5
422.5	391.25
432.5	390.5
426.25	380.75
420.5	395.5
412.5	333.0
384.5	352.5

Fig. 1B

AL	IF
892.75	691.75
844.00	671.50
784.25	795.50
769.25	673.00
765.25	723.75
950.00	782.50
895.00	767.50

Fig. 1C

AL	IF
9.353123	2.312974
7.671801	6.142963
10.137070	6.411062
9.977250	5.423477
9.049331	6.701209
7.584210	4.833866
8.142458	5.543974

Fig. 2

	State 3		State 4	
	**AL**	**IF**	**AL**	**IF**
**Brain**	126.3499	118.1482	14.19844	11.22068
	108.54	113.3641	12.16952	10.79912
	100.738	109.8192	10.32764	15.9358
	98.31572	101.2775	9.32304	15.09504
	119.3349	94.90312	12.55584	10.23872
	83.59504	103.9765	10.3182	13.32268

**Heart**	405.0075	337.3834	60.22433	84.04774
	483.6888	452.5117	93.97011	46.02583
	251.1381	374.5365	46.00263	62.32952
	142.1575	300.876	44.62238	45.09197
**Muscle**	212.2639	137.5318	51.0968	45.25544
	103.7866	123.8094	42.17336	41.54104
	162.7558	246.3251	28.9268	37.13176
	137.7334	140.5392	34.25536	29.12072
**Liver**	26.4002	35.27042	5.4352	5.96838
	19.03132	39.15828	3.88256	8.16972
	17.64486	42.85346	3.5562	6.01944
	33.02708	45.80884	6.22878	7.38232
	28.30482	44.63742	6.83088	7.51174
	25.77784	33.8812	4.33422	5.36672

Fig. 2D insert

**AL**	**IF**
4.857264	5.909547
4.901745	4.793099
4.961718	7.119177
5.302335	6.205209
4.143656	5.942354
5.947515	6.313204

Fig. 3 A-B

	**State 3**		**State 4**	
	**AL**	**IF**	**AL**	**IF**
**Succinate**	58.68074	77.70864	12.56056	17.32828
	41.55026	115.0570	9.305100	19.67902
	74.14678	88.53984	14.93538	15.86126
	38.28100	85.37026	7.145600	16.25540
	68.05504	119.1456	13.01462	22.28256
	71.52476	71.08026	11.29468	14.51066
**TMPD**	38.21414	65.29432	28.4744	49.6176
	35.04338	77.74434	24.6832	51.93020
	48.10534	62.68464	35.13744	44.87482

Fig. 3C

COX-IV

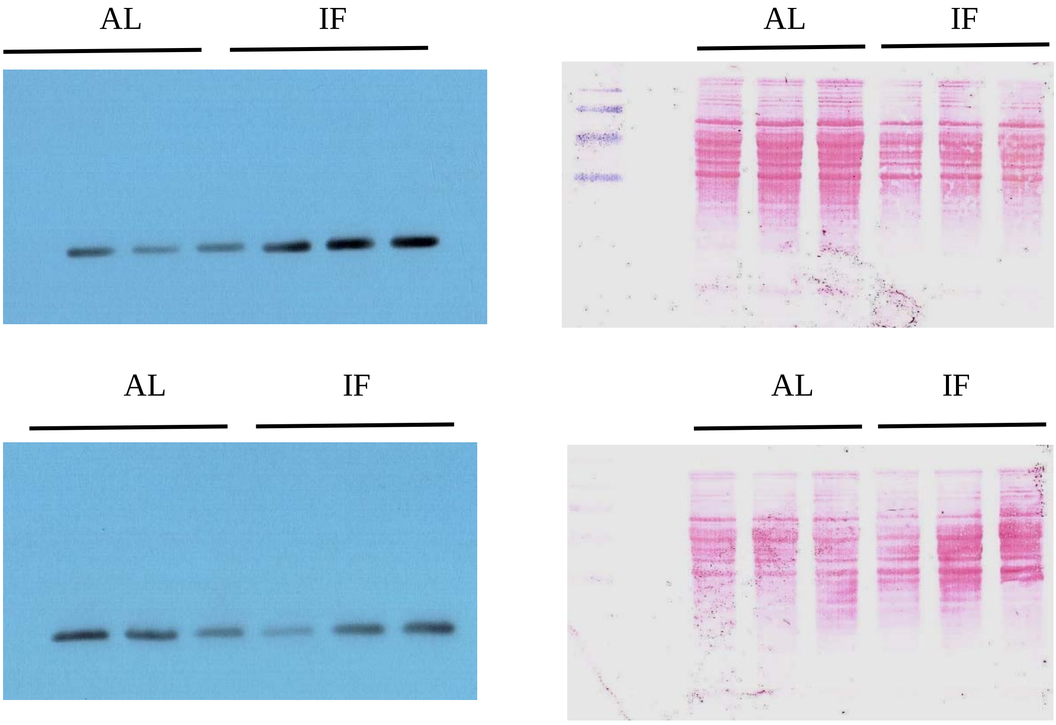

NRF-1

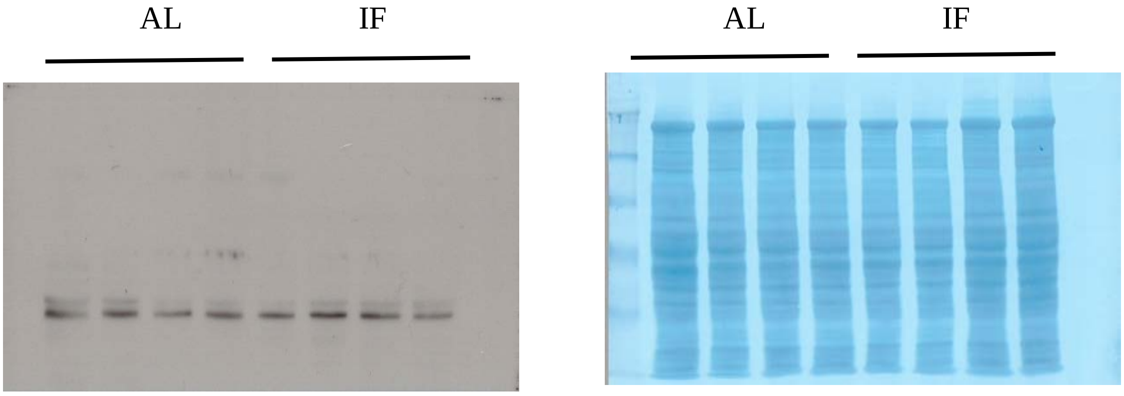

COX-IV

**AL**	**IF**
0.744299	2.299761
0.20831	2.771185
0.350921	3.294644
3.168671	*0.783047*
1.237715	1.931862
2.458755	1.361634

NRF-1

AL	IF
1.262878	0.669002
1.269339	1.120377
0.675567	1.132139
0.792216	0.799791

Fig. 3D

AL	IF
1.8649	1.792077
1.740566	1.828337
1.585023	1.479375
1.595124	1.622394

Fig. 4A

	AL	IF
**Brain**	0.11185	0.10039
	0.10606	0.08608
	0.09418	0.11385
	0.08287	0.10502
	0.08775	0.09038
	0.09934	0.08966
**Heart**	0.21657	0.211217
	0.185937	0.242192
	0.180516	0.212095
	0.122467	0.175726
**Muscle**	1.855056	1.698024
	2.64941	1.914408
	1.857794	2.273353
	2.148001	2.801651
**Liver**	0.690503	0.890425
	0.871025	0.935959
	0.676401	0.919982
	0.606456	1.239641
	1.015853	0.702176
	0.78452	0.77899
	0.684225	0.981545
		0.852108

Fig. 4B

	AL	IF
**Brain**	0.09	0.08
	0.10	0.08
	0.09	0.10
	0.08	0.10
	0.07	0.10
	0.12	0.09
**Heart**	0.05	0.06
	0.04	0.05
	0.07	0.06
	0.09	0.06
**Muscle**	0.9	1.2
	2.6	1.5
	1.1	0.9
	1.6	2
**Liver**	2.6	2.5
	4.6	2.3
	3.4	2.9
	3.1	1.5
	2.8	2.2
	2.7	2.5

Fig. 5A

Brain

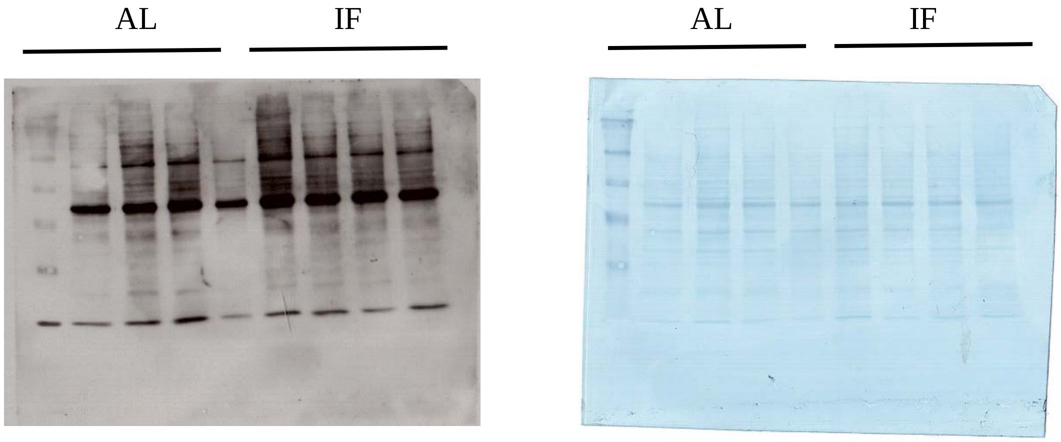

Heart

AL IF

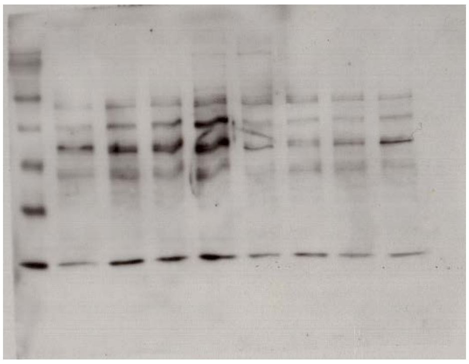

AL IF

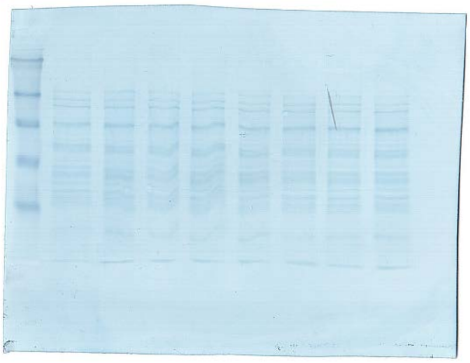

Skeletal Muscle

AL IF

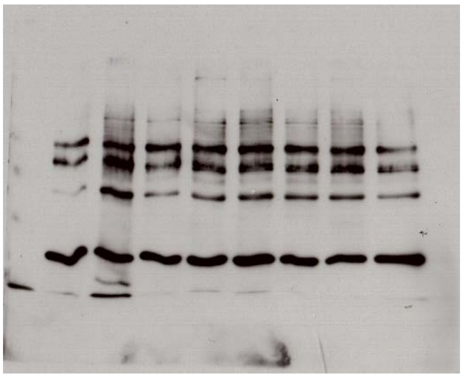

AL IF

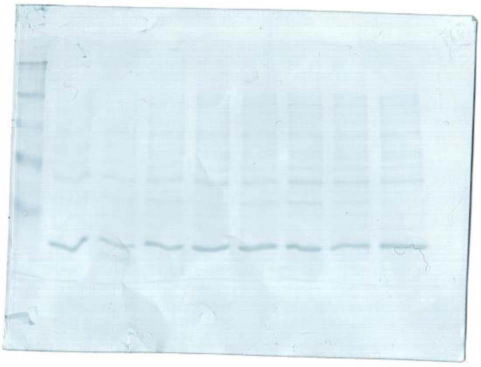

Liver

AL IF

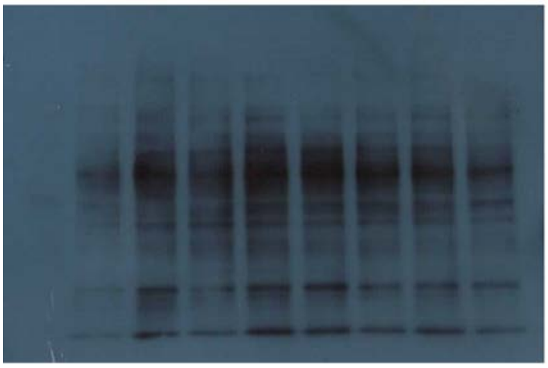

AL IF

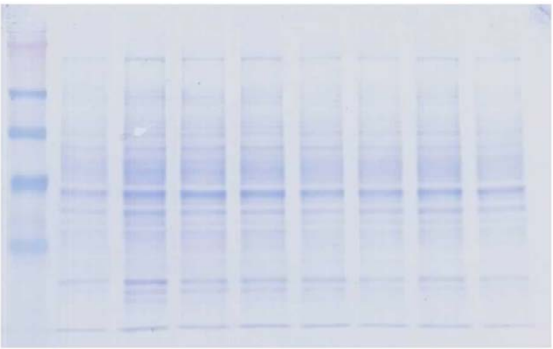

	AL	IF
**Brain**	0.665377	1.619697
	1.224928	1.999759
	1.363456	1.597181
	0.746239	1.368806
**Heart**	0.704948	0.715059
	0.976629	0.514308
	0.922783	0.637624
	1.39564	0.617034
**Muscle**	0.768227	1.123662
	1.274156	1.000853
	0.893812	1.110795
	1.063805	0.984933
**Liver**	0.619559	1.717729
	0.911207	1.905121
	1.048229	1.482572
	1.421005	1.859742

Fig. 5B

	AL	IF
**Brain**	0.087372	0.066529
	0.050837	0.078721
	0.071732	0.072294
	0.068798	0.082377
**Heart**	0.023187	0.012619
	0.016401	0.018229
	0.017737	0.010358
	0.019408	0.011067
	0.020379	0.012111
**Muscle**	0.015934	0.010085
	0.013541	0.016352
	0.012706	0.020643
	0.010945	0.0201
	0.012746	0.01961
**Liver**	0.020083	0.018525
	0.012504	0.026394
	0.015059	0.014808
	0.024566	0.017158

Fig. 5C-D

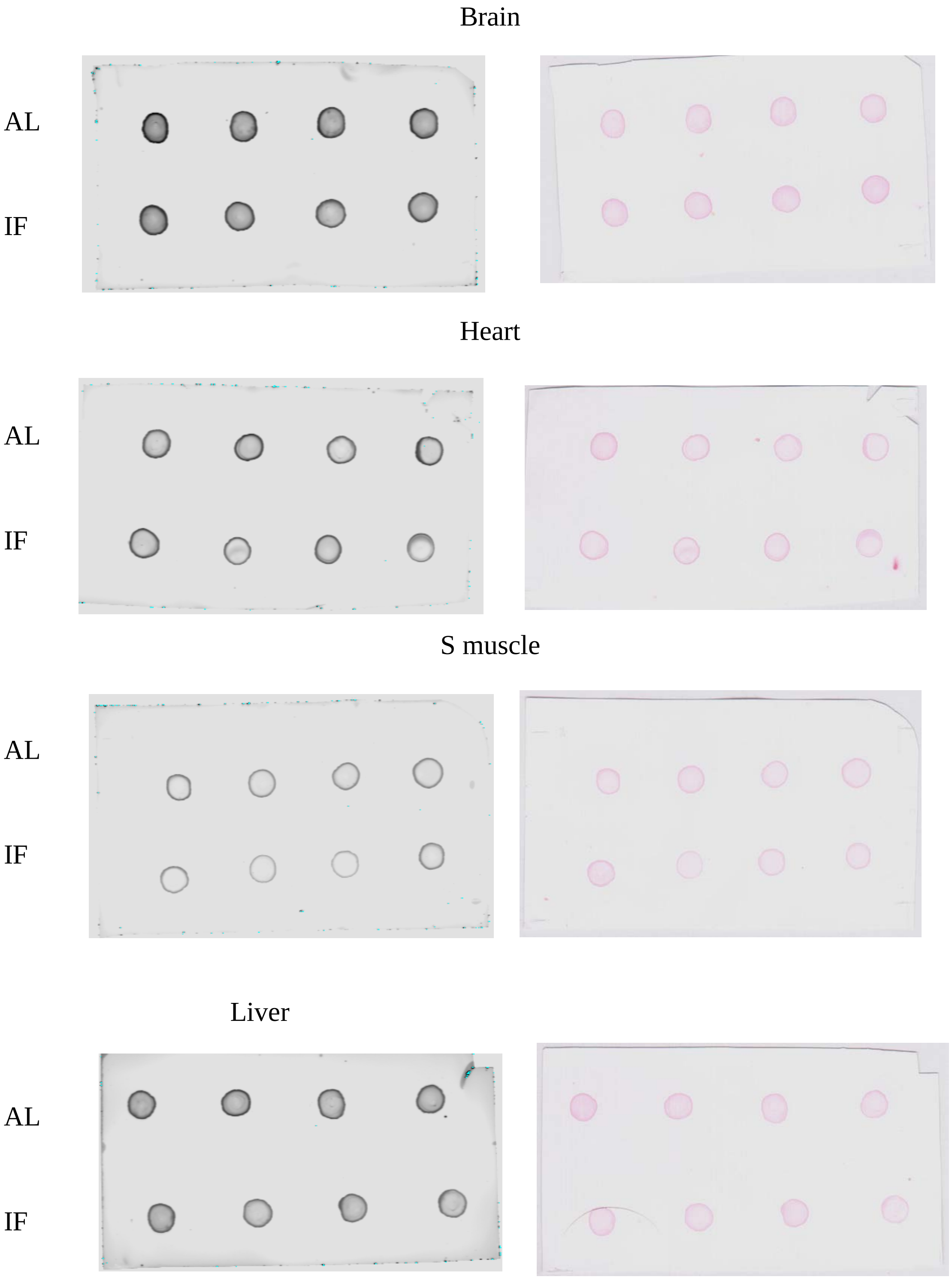

	AL	IF
**Brain**	1.18	1.00
	0.96	0.81
	0.94	0.68
	0.93	0.74
**Heart**	0.92	1.06
	1.16	0.67
	0.83	0.93
	1.09	0.70
**Muscle**	1.03	0.68
	0.84	0.50
	0.98	0.40
	1.15	1.03
**Liver**	0.98	0.99
	1.02	0.75
	0.96	0.84
	1.03	0.81

Table 1

Glutathione Peroxidase

	AL	IF
**Brain**	24.48266	19.99656
	24.08705	21.6548
	18.85795	19.4528
	23.17524	23.56511
**Heart**	141.2655	98.75057
	131.6445	95.8475
	107.7561	66.52503
	98.84933	104.7313
**Muscle**	203.9596	185.7143
	232.8571	172.6091
	251.9017	195.0758
	264.2765	226.6100
**Liver**	214.8002	161.7345
	222.4070	158.4052
	128.7111	178.2600
	107.9963	200.6118

Glutathione Reductase

	AL	IF
**Brain**	5.564513	5.287010
	5.322423	5.617791
	4.78907	5.365539
	5.176286	5.663179
**Heart**	5.450696	4.671971
	4.064444	4.469462
	3.928849	3.184844
	4.621091	3.822166
**Muscle**	12.38839	11.38342
	13.42114	12.09044
	13.76252	12.47525
	11.61017	13.98422
**Liver**	11.96721	11.67002
	12.15407	12.41364
	11.13420	11.48392
	12.28840	12.53030

Catalase

	AL	IF
**Brain**	0.312	0.244
	0.290	0.232
	0.241	0.216
		0.225
**Heart**	1.430	1.203
	0.667	0.417
	0.964	1.030
	0.670	0.751
**Muscle**	0.810	0.719
	0.714	0.480
	0.944	0.945
	0.896	0.807
**Liver**	5.287	5.308
	4.198	5.815
	5.422	4.675
	4.061	4.018

Table 2

Total GSH

	AL	IF
**Brain**	34.60019	49.11647
	47.39972	47.27686
	37.02807	47.3427
	44.80668	59.03716
**Heart**	21.51806	23.07065
	22.85665	20.9627
	19.35153	23.85067
**Muscle**	43.88334	88.9047
	76.60027	58.44491
	74.77686	118.2952
	78.17197	56.77978
**Liver**	12.14321	16.21222
	11.06292	16.10899
	10.88948	13.58537

GSH

	AL	IF
**Brain**	28.15035	44.89362
	38.45729	38.99266
	30.68401	38.29562
	42.95668	48.53835
**Heart**	14.31335	20.45201
	17.82626	17.60174
	11.57087	20.25429
**Muscle**	20.66204	66.17532
	42.30978	34.17741
	59.62256	85.76438
	55.44108	29.69962
**Liver**	10.48447	13.12573
	8.675137	13.13434
	7.855144	11.83606

GSSG

	**AL**	**IF**
**Brain**	3.224915	4.142099
	4.471217	4.523539
	3.172027	5.249402
**Heart**	3.602353	1.309317
	2.515195	1.680476
	3.890332	1.798188
**Muscle**	11.61065	11.36469
	17.14524	12.13375
	7.577147	16.26541
	11.36545	13.54008
**Liver**	0.829372	1.543247
	1.193892	1.487327
	1.517169	0.874654

GSH/GSSG

	**AL**	**IF**
**Brain**	8.729023	9.413744
	8.601079	8.465855
	9.673313	9.246454
**Heart**	3.973334	15.62037
	7.087427	10.47426
	2.974262	11.26372
**Muscle**	1.779576	5.82289
	2.467727	2.816722
	7.868734	5.272808
	4.878039	2.193459
**Liver**	12.64145	8.505269
	7.266263	8.830834
	5.177500	13.53228
